# Review: Human stem cell-based 3D in vitro angiogenesis models for preclinical drug screening applications

**DOI:** 10.1007/s11033-023-09048-2

**Published:** 2024-02-01

**Authors:** Aibhlin Esparza, Nicole Jimenez, Edgar A. Borrego, Shane Browne, Sylvia L. Natividad-Diaz

**Affiliations:** 1 Department of Metallurgical, Materials, and Biomedical Engineering (MMBME), The University of Texas at El Paso (UTEP), El Paso, TX USA; 2 3D Printed Microphysiological Systems Laboratory, The University of Texas at El Paso, El Paso, TX USA; 3 Department of Anatomy and Regenerative Medicine, Tissue Engineering Research Group, Royal College of Surgeons, Dublin, Ireland; 4 CÚRAM, Centre for Research in Medical Devices, University of Galway, Galway, H91 W2TY Ireland; 5 Border Biomedical Research Center, University of Texas at El Paso, El Paso, TX USA

**Keywords:** hiPSCs, Angiogenesis, 3D tissue models, Drug screening

## Abstract

Vascular diseases are the underlying pathology in many life-threatening illnesses. Human cellular and molecular mechanisms involved in angiogenesis are complex and difficult to study in current 2D in vitro and in vivo animal models. Engineered 3D in vitro models that incorporate human pluripotent stem cell (hPSC) derived endothelial cells (ECs) and supportive biomaterials within a dynamic microfluidic platform provide a less expensive, more controlled, and reproducible platform to better study angiogenic processes in response to external chemical or physical stimulus. Current studies to develop 3D in vitro angiogenesis models aim to establish single-source systems by incorporating hPSC-ECs into biomimetic extracellular matrices (ECM) and microfluidic devices to create a patient-specific, physiologically relevant platform that facilitates preclinical study of endothelial cell-ECM interactions, vascular disease pathology, and drug treatment pharmacokinetics. This review provides a detailed description of the current methods used for the directed differentiation of human stem cells to endothelial cells and their use in engineered 3D in vitro angiogenesis models that have been developed within the last 10 years.

## Introduction

Pathological angiogenesis and vascular diseases are the underlying comorbidities in many life-threatening illnesses. The pathological growth and proliferation of blood vessels termed the “activated angiogenic switch”, is one of the six hallmarks of tumor growth and cancer progression [1, 2]. Cardiovascular disease refers to damaged, narrowed, or blocked blood vessels composed of dysfunctional endothelial cells that can cause stroke or myocardial infarction [3, 4]. Cardiovascular Diseases (CVD) including atherosclerosis, myocardial infarction, stroke, peripheral artery disease, and venous disease collectively remain the leading causes of death in the United States [5]. Additionally, the World Health Organization cites CVD as the leading cause of death worldwide and expects the death rate to continue rising within the next 15 years regardless of socioeconomic background [6].

Although angiogenesis is an underlying mechanism for the progression of many diseases and tissue repair, it is a complicated, multi-factorial process that necessitates further investigation to fully understand its role in tissue regeneration and disease. The complex processes involved in angiogenesis and relatively expensive in vivo animal models which require advanced technical skills make it difficult to evaluate the efficacy, toxicity, and pharmacokinetics of candidate drugs in preclinical studies [7–10]. In vitro models with human pluripotent stem cells provide a less expensive, more controlled, and reproducible platform for better quantification of isolated angiogenic processes in response to external chemical or physical stimulus [9–12].

Current 3D in vitro angiogenesis models incorporating stem cell-derived endothelial cells with engineered biomaterials as extracellular matrix (ECM) have provided important insight into complex therapeutic and pathological angiogenesis [11, 13]. In particular, the use of human induced pluripotent stem cell (hiPSC) derived vascular lineage cells, such as endothelial cells and pericytes, have many advantages such as reducing patient source variation along with increasing the genetic diversity of test samples so that patients from different backgrounds (gender, age, race/ethnicity, disease state, etc.) are represented in these studies [12]. Furthermore, hiPSCs can theoretically provide an endless supply of patient-specific, vascular cells which enables in vitro models to be used for the research of physiological or pathological angiogenesis, vascular diseases, drug toxicity, and regeneration of ischemic or damaged tissues [13, 14].

Several methods to differentiate stem cells to endothelial cells have been established within the last decade. These protocols have evolved from spontaneous embryoid body formation at the pluripotency stage to directed differentiation of 2D pluripotent stem cell monolayers with small molecules and growth factors. The latter methods modulate the Wnt, SMAD, and MEK/MAPK pathways to recapitulate the differentiation and specification of endothelial cells during developmental vasculogenesis in vitro [7, 10, 15, 16]. Toward this end, significant progress has been made in directed differentiation protocols for human pluripotent stem cell monolayers to the endothelial cell (EC) lineage and their incorporation into 3D in vitro angiogenesis models to obtain physiologically-relevant data. Current EC differentiation protocols have improved total differentiation time, expression of mature endothelial cell markers, and implementation of efficient small-molecules to culture media for a single differentiation. Most protocols consist of a 5–10 day differentiation process with an additional maturation phase that can last up to 60 days before the ECs are used in a functional assay [17–22]. Additionally, many of these studies have observed the expression of mature endothelial cell markers such as CD31 and VE-Cadherin at Day 5–7 or after the maturation phase (Day 12–15) [17–22]. Current differentiation protocols are diverse and entail the use of different media formulations from the pluripotency stage to the differentiation and maturation process [7, 16–23]. Considering the culture and maintenance of the pluripotent stem cells before differentiation, the total process can take 2 weeks or up to 3 months with 2–3 different media formulations to obtain ECs at various maturity levels and different sub-types (arterial vs. venous). Current studies to develop 3D in vitro angiogenesis models aim to establish single-source systems by incorporating hiPSC-ECs into biomimetic extracellular matrices (ECM) and microfluidic devices to create a patient-specific, physiologically relevant platform that facilitates preclinical study of endothelial cell-ECM interactions, vascular disease pathology, and drug treatment pharmacokinetics. Data output from 3D in vitro angiogenesis models relevant to human physiology include dose–response curves with EC50/IC50 values, capillary-like network formation, and variation in lineage-specific biomarker expression in response to biochemical or biophysical stimulus. This review provides a detailed description of the current methods used for the directed differentiation of human stem cells to endothelial cells and their use in engineered 3D in vitro angiogenesis models that have been developed within the last 10 years.

## Current endothelial cell differentiation methods from human pluripotent stem cells

Several endothelial cell differentiation methods have been developed within the last decade. These protocols have evolved from spontaneous embryoid body formation to directed differentiation of 2D pluripotent stem cell monolayers with small molecules and growth factors. The latter methods modulate the Wnt, SMAD, and MEK/MAPK pathways to recapitulate developmental vasculogenesis during the differentiation and specification of endothelial cells in vitro [7, 8, 10, 16, 17, 20, 22, 24–32]. Table 1 summarizes some of the current protocols and methods for direct differentiation of human pluripotent stem cells to endothelial cell lineage.

**Table 1:** Summary of current protocols to differentiate human pluripotent stem cells to endothelial cells

Citation	Length of differentiation protocol	Cells	Media	Yield (%)
Kusuma et al. [17]	12 day	hiPSC to early vascular lineage cells (EVCs)	• 6 days alpha-MEM medium + 10% FBS, mM beta-mercaptoethanol • On day 6, ECGM with 2% FBS, 50 ng/mL VEGF, 10 uM SB431542 for another 6 days • VE-CAD + cells ECGM + 50 ng/mL NEGF + 10 uM SB431542 for another 6 days	yielded 8–25% VCAD + EVCS
Orlova & Mummery [21]	10 day	hiPSC to endothelial cells	• BMP4, activin A, CHIR, VEGF in BP(E)L medium composed of IMDM, Ham’s F12, PFHMII, 10% BSA, Lipids (100x), ITS-X (100x), αMTG (13 µl in 1 ml IMDM), AA2P (5 mg/mL), GlutaMAX (200 mM), and Pen-strep (5000 U/mL) • After 3 days, vascular specification medium with BP(E)L + VEGF + SB431542	yielded ~ 20% CD31 + /VCAD + ECs
Prasain and Yonder [22]	21 day	hiPSCs to endothelial colony forming cells (ECFCs)	• mTESR1 medium for 2 days • addition of activin A (10 ng/ml) in FGF-2, VEGF-A165 and BMP4 (10 ng/ml) for 24 h • Next day, Stemline II complete media with FGF-2, VEGF165, and BMP4 • Day 12, 50% EGM-2 and 50% complete Stemline II differentiation media for 7 days	NRP-1 + CD31 + cells gave rise to 60% more endothelial colonies
Lian and Pelacek [18]	5 day	hiPSCs to endothelial progenitor cells (EPCs)	• 2 days with 6–10 uM CHIR99021 in LaSR basal medium with Advanced DMEM/F12, 2.5 mM GlutaMAX, and 60 mg/ml ascorbic acid • 3–4 days LaSR basal medium without CHIR99021	Day 5 55% CD34 + /CD31 + and 57% KDR + EPCs Day 60, 99.2% of the purified population expressed CD31 and 98.9% was VCAD +
Bao and Pelacek [19]	5 day	hiPSCs to endothelial cell progenitor cells (EPCs)	• Day 0, DMEM + 3–9 μM CHIR9902 + 100 μg/mL ascorbic acid • After 2 days, DMEM + ascorbic acid without CHIR99021 for 3 to 4 days • EGM-2 medium or human endothelial- SFM + 20 ng/mL bFGF + 10 ng/mL EGF for 10 additional days • At Day 15, the ECs supplemented with EGM-2 + 50 ng/mL VEGF	At Day 5, the population expressed 20% to 32% CD34/31 + and 29% CD144 +
Zhang, Chu, et al. [32]	6 day	hiPSCs to arterial endothelial cells (AECs)	• Day 0, E8BAC media (E8 media supplemented with 10 μM Y27632 • For 2 days, E8BAC media (E8 media supplemented with 5 ng/mL BMP4, 25 ng/mL Activin A, and 1 μM CHIR99021) • Day 2 to day 6, E5 media supplemented with 100 ng/mL FGF2, 50 ng/mL VEGFA, 10 µM SB431542, 5 µM RESV, 5 µM L690 (Five Factor Protocol)	After 6 days, 44–90% purity
Harding et al. [20]	8–10 day	hiPSCs/hESCs to endothelial cells	• Day 0, MEF conditioned hESC medium + 10 ng/mL of FGF2 • After 1 day, StemDiff APEL medium + 6 uM CHIR99021 for 2 days • StemDiff APEL medium + 25 ng/mL, BMP4 + 10 ng/mL FGF2 + 50 ng/mL VEGF for another 2 days • “Peripheral cells” supplemented with EC Growth Medium MV2 (ECGM-MV2) + additional 50 ng/mL VEGF	• On day 4, 67.8% CD31 + /CD144 + , the “peripheral cells” were 84% CD31 + /CD144 + and the “central cells” were 55.6% CD31 + /CD144 + • 94%-97% CD31 + , 73%-81% CD34 + , and 78% to 83% CD144 • After one passage the ECs expressed 99.7% CD31 + and 96.i% CD144 +
Natividad-Diaz and Healy et al. [35]	5 day	hiPSCs to CD31 + endothelial cell population	• Day -2, Essential 8 Medium supplemented with 10 uM ROCK inhibitor (day -2) • Day -1, medium replaced with Essential 8 without ROCK inhibitor • Day 0, Essential 8 Medium supplemented with 6 uM CHIR 99021 • Day 2, addition of Essential 8 Medium supplemented with 10 ng/ml BMP4, 50 ng/ml VEGF-A_165_, and 10 uM SB431542 • E8BVi every day for 3 more days up to the final stage of differentiation on day 5	• Day 5, 33% CD31 + , 58% VEGFR2 (KDR) + , and 68% VE-CAD • Overall differentiation efficiency for CD31 expression = 27.8% ± 4.6% • Purified population after two passages was 92% CD31 + , 78% VEGFR2 (KDR) + , and 99% VECadherin +
Ohshima, Kamei et al. [93]	9 day	hiPSCs to individual brain microvascular endothelial cells (iBMECs)	• iPSCs were cultured in Essential 8 Flex Medium i • Day 1, E8 was replaced with unconditioned medium (UM) + 20% KnockOut Serum Replacement + 1 X MEM nonessential amino acids + 1 mM L-glutamine + 0.1 mM β-mercaptoethanol • UM was refreshed every day from D1-D4 • Cells cultured in human endothelial cell medium + 1% platelet-poor plasma (PPP) serum + 20 ng/mL basic fibroblast growth factor + 10 μM retinoic acid from D5-D8 • Medium was replaced with EMC minus bFGF on D9	hPPP improved the differentiation efficiency compared to bPPP (100% in hPPP vs. 66.7% in bPPP)
Rosa et al. [33]	20 days	hiPSCs into arterial and venous ECs	• Undifferentiated cells cultured with chemically defined medium (CDM) + 10 ng/ml + BMP4 + 20 ng/ml FGF-$\beta$ for 1.5 days • 50 ng/ml BMP4 + 20 ng/ml FGF-$\beta$ until day 5 • Media containing VEGF + T $\beta$ 4 from day 5 to day 10 • VEGF (50 ng/ml) for 4 passages for arterial endothelial like cells (AELCs) • VEGF (10 ng/ml) for 4 passages for venous endothelial like cells (VELCs)	From 1 million undifferentiated cells, obtained ~ 7.5 million AELCs and VELCs after 20 days of differentiation

Kusuma et al. developed a 12-day monolayer differentiation protocol for hPSCs to early vascular lineage cells (EVCs) with a 6-day maturation phase to endothelial cell lineage [17]. This consisted of culturing the hPSCs for 6 days in alpha-MEM medium supplemented with FBS (fetal bovine serum) and β-mercaptoethanol. Subsequently, differentiated cells were grown for 6 days in endothelial cell growth media (ECGM) supplemented with FBS, vascular endothelial cell growth factor (VEGF), and SB431542. VEcad^+^ cells were then sorted by MACS and cultured for 6 days using the same conditions. In addition, a serum-free alternative for EVC differentiation is reported consisting of culturing the hPSCs with alpha-MEM with Knockout serum replacement, β-mercaptoethanol, non-essential amino acids, and L-glutamine for 6 days, followed by 6 days of culturing with ECGM with VEGF, SB431542, knockout serum replacement, β-Mercaptoethanol, essential amino acids, and glutamine. This yielded 8–25% VCAD + EVCS which continued to EC maturation. No subpopulation of Day 12 EVCs were mature ECs since there was no detection of CD31, lectin, eNOS, VWF, or Ac-LDL uptake. Therefore, additional culture was needed to mature the EVCs to endothelial cell lineage. EVCs exhibited typical membrane expression of VE-cad and CD31, lectin binding, cytoplasmic expression of eNOS and vWF, and network formation after culture them on Matrigel. Culture of EVCs and matured ECs forms networks in collagen and completely synthetic hyaluronic acid (HA)-based hydrogel. In addition, EVCs were able to form networks when encapsulated within collagen gels while sorted VE-cad + or VE-cad- cells individually were not. EVCs that were cultured in a hyaluronic acid hydrogel demonstrated multicellular networks by Day 3. Finally, an in vivo Matrigel plug assay also revealed EVCs anastomosed with perfused murine host microvasculature and generated human-only microvascular structures.

A 10-day SMAD pathway-based endothelial differentiation protocol was reported by Orlova et al.[21] Mesoderm specification was induced in hPSCs by adding bone morphogenetic protein 4 (BMP4), activin A, small-molecule inhibitor of glycogen synthase kinase-3β (CHIR), and vascular endothelial growth factor (VEGF) in BP(E)L medium composed of IMDM, Ham’s F12, PFHMII, BSA, Lipids, ITS-X (insulin-transferrin-selenium-ethanolamine), αMTG (mono thiol glycerol, in IMDM), AA2P, GlutaMAX, and Pen-strep. After 3 days, cells were cultured in a vascular specification medium that consisted of BP(E)L supplemented with VEGF and the transforming growth factorβ (TGFβ) pathway small-molecule inhibitor SB431542 (SMAD signaling pathway). The protocol yielded ~ 20% CD31 + /VCAD + ECs which were isolated on Day 10. It was demonstrated that ECs form functional blood vessels through an in vivo assay with a zebrafish xenograft. Additionally, it was found that hPSC-derived ECs anastomosed with the host vasculature and performed better than human umbilical vein endothelial cells (HUVECS).

Differentiation of pluripotent stem cells to endothelial colony-forming cells (ECFCs) in a 21-day protocol has also been previously reported [22]. Initially, hPSCs were culture in mTESR1 media for 2 days. After, activin A in the presence of FGF-2, VEGF-A165, and BMP4 was added for 24 h to direct cultures toward the mesodermal lineage. The next day, cells were cultured in Stemline II complete media containing FGF-2, VEGF165, and BMP4 to promote endothelial cell specification and proliferation by using the VEGF-KDR signaling pathway. On Day 12 the CD31 + /NRP1 + cells were sorted and cultured in 50% EGM-2 and 50% complete Stemline II differentiation media to generate ECFCs. After 2 days, the media was replaced with three parts of EGM-2, and one part of differentiation media was added to the cultures for seven days. The hiPSC-ECFCs were encapsulated in collagen gels and implanted subcutaneously in an immunodeficient mouse to test their angiogenic potential. After 14 days, the implanted ECFCs formed durable and functional in vivo human vessels in the subcutaneous pouch.

Human induced pluripotent stem cells (iPSCs)-derived EC precursor cells were differentiated into arterial and venous-like ECs in chemically defined conditions by modulating the VEGF pathway [33]. hiPSCs were differentiated into mesoderm progenitors (Brachyury pos) and differentiated into endothelial precursor cells (EPCs) that expressed venous and arterial markers by culturing cells in media containing VEGF and TB4 from day 5 to day 10. The percentage of cells expressing CD31 and KDR increased over time (at least for 10 days). Based on the EC expression profile, CD31 + cells were isolated as EPCs. To obtain arterial endothelial-like cells (AELCs), EPCs were cultured in an EC-serum-free medium supplemented with a high concentration of VEGF for passage 0 to passage 4 (P0 to P4), and at P4, the iPSC-derived ECs expressed similar or higher mRNA levels of CD31, VE-cadherin, and KDR than human umbilical artery endothelial cells (HUAECs). Cells expressing EC markers, arterial markers, and EC functional activity, were named AELCs. To obtain venous endothelial-like cells (VELCs), EPCs were cultured in EC-serum-free medium supplemented with a low concentration of VEGF for 4 passages. iPSC-derived ECs at P4 expressed similar mRNA levels of CD31, KDR, and VE-cadherin than HUVECs. In general expression of EC markers, expression of arterial markers, and EC functional activity, the cells were named as AELCs. The results showed that EPCs cultured in an EC-serum–free medium supplemented with a high concentration of VEGF gave rise to AELCs. After 20 days of differentiation, 7.5 million AELCs and VELCs were obtained from 1 million undifferentiated iPSCs. Scaffolds based on stable ultrathin polymeric nanofilms prepared from polymers including poly (L-lactic acid) (PLLA) and poly (caprolactone) (PCL) and poly(dimethylsiloxane) (PDMS) were used to create a nanofilm and placed into cell culture inserts. iPSC-derived AELCs and VELCs were cultured for 72 h on top of the nanofilms. The cells were able to form a monolayer and expressed VE-cadherin and ZO-1 a significantly higher VE-cadherin intensity for both VELCs and AELCs cultured for 72 h in PCL nanofilms compared to those cultured in PDMS nanofilms.

Lian and Palecek developed a 5-day protocol to produce endothelial progenitor cells (EPCs) from human pluripotent stem cells which could then be further matured to tube-forming ECs (seen at Day 60) using the WNT/$\beta$-catenin signaling pathway [18]. The differentiation protocol was initiated by treating the cells for 2 days with CHIR99021 in LaSR basal medium consisting of Advanced DMEM/F12, GlutaMAX, and ascorbic acid. The cells were then maintained in LaSR basal medium without CHIR99021 for 3–4 more days. On Day 5 the population consisted of 55% CD34 + /CD31 + and 57% KDR + EPCs. The hPSC-derived endothelial progenitors and endothelial cells identified with WNT/β-catenin signaling pathway. To differentiate the EPCs to mature endothelial cells, the Day 5 CD34 + cells were magnetically sorted and replated on collagen-IV-coated dishes in EGM-2 and split every 4–5 days. On Day 60, 99.2% of the purified population expressed CD31 and 98.9% was VCAD + . Additionally, the purified day 60 endothelial cells demonstrated Ac-LDL uptake and tube-forming ability in a Matrigel angiogenesis assay supplemented with an additional VEGF treatment. Bao and Palecek developed a 5-day chemically-defined albumin-free protocol for the differentiation of human pluripotent stem cells to endothelial progenitor cells with an additional 10-day endothelial cell differentiation/maturation stage via modulation of WNT signaling [19]. On day 0, the cells were fed DMEM supplemented with CHIR99021 (Selleckchem) and ascorbic acid. After 2 days, cells were cultured in DMEM supplemented with ascorbic acid without CHIR99021 for 3 to 4 days. On Day 5, the population expressed 20% to 32% CD34/31 + and 29% CD144 + . On Day 5, the differentiated populations were purified based on CD34 expression, using the Wnt signaling pathway. For endothelial cell differentiation, the purified CD34 + cells were plated on collagen IV-coated dishes and fed EGM-2 medium or human endothelial- SFM supplemented with bFGF and EGF for 10 additional days. On Day 15, the endothelial cells demonstrated Ac-LDL uptake and tube formation on a 24-h Matrigel angiogenesis assay consisting of a 24-well tissue culture plate pre-coated with Matrigel with EGM-2 supplemented with VEGF.

The ability to form functional arterial endothelial cells (AECs) from human induced pluripotent stem cells (hiPSCs) provides a model for vascular disease in vitro [34]. Zhang et al. were able to transplant properly specified AECs, hypothesizing this will improve arteriogenesis and formation of ischemic tissues compared to generic endothelial cells and pluripotent stem cell-derived vascular progenitors. VEGF, WnT, and NOTCH signaling pathways were used for the AEC differentiation. To evaluate the function of candidate factors in human AEC differentiation, a human embryonic stem cell reporter line during CRISCPR-Cas9 technology was created to target EFNB2 with tdTomato and EPHB4 with EGFP, markers that have been widely used for AECs and venous endothelial cells (VECs). AECs were generated using the Five Factor protocol. The function of the hiPSCs in a mouse model of myocardial infarction was completed to detect how the AECs and VECs contributed to the arterial and venous structures, respectively. When evaluating the candidate factors for arteriovenous specification, it was demonstrated that VEGFA, WNT3A, RESV, FGF2, LFL, L690, and SB431542 promoted AEC differentiation, whereas insulin, BMP4, and PDGF-BB inhibit AEC differentiation. The combination of FGF2, VEGFA, SB431542, RESV, and L690 (the “five factor”) in the absence of insulin greatly improved AEC differentiation. The functional properties of AECs characterized an arterial phenotype distinct from human umbilical vein endothelial cells (HUVECs) and VECs. The function of the hiPSCs in a mouse model demonstrated that AECs contributed to the host arterial structures whereas VECs contributed to the venous structure. The AECs generated in this study were able to significantly enhance survival rates in a mouse myocardial infarction model. This study provided insight into human arterial development and differentiation and will facilitate the understanding of the underlying mechanisms of vascular disease. Most recently, Harding and colleagues developed an 8–10 day endothelial cell differentiation protocol [20]. In this study, the hiPSC/hESCs were grown on mouse embryonic fibroblast feeder (MEF) layers, and small clusters were manually passaged onto Matrigel-coated plates in MEF conditioned hESC medium supplemented with FGF2. After 1 day, the culture medium was switched to StemDiff APEL medium with CHIR99021 for 2 days. The cells were then cultured in StemDiff APEL medium supplemented with BMP4, FGF2, and VEGF for another 2 days. On day 4, the culture self-assembled into a monolayer of cells with cobblestone morphology, termed “peripheral cells”, with sporadic clusters of smaller cells, termed “central cells”. The signaling pathways consisted of Wnt and then VEGF, BMB, and FGF signaling. The “peripheral cells” were released and replated with EC Growth Medium MV2 (ECGM-MV2) supplemented with an additional VEGF. The medium was changed every 2 days for 4–6 days to generate mature ECs that were 94%–97% CD31 + , 73%–81% CD34 + , and 78% to 83% CD144 + . After one passage the ECs expressed 99.7% CD31 + and 96.i% CD144 + . The hiPSC-ECs were subcutaneously injected with a Matrigel plug in the hind limb of an immune deficient NSG mouse. After 2 weeks, immunostaining for human CD31 of the explanted plug revealed vessels and branches. The hiPSC-ECs were then injected into a hind limb ischemia mouse model. A tail vein injection of FITC-Dextran ten weeks after transplantation revealed some tube formation and localization of human ECs but the ischemic area remained visible.

Natividad-Diaz and Healy et al. developed a refined 5-day method for obtaining a CD31 + endothelial cell population from hiPSCs that required the use of a chemically defined basal medium from the pluripotency stage to the final stage of differentiation and did not require a maturation period [35]. In this modified protocol, human pluripotent stem cells were passaged onto plates coated with growth factor-reduced Matrigel and fed Essential 8 (E8) Medium supplemented with ROCK inhibitor (day-2). The medium was replaced the next day (day-1) with E8 without ROCK inhibitor. The cells were grown to approximately 80% confluency and differentiation was initiated on day 0, by feeding the cells E8 supplemented with CHIR 99021. Endothelial lineage specification started on day 2 with the addition of E8 Medium supplemented with BMP4, VEGF-A_165_, and SB431542. The cells were fed this medium (E8BVi) every day for 3 more days up to the final stage of differentiation on day 5. After day 5, the cells were magnetically sorted for CD31 + expression and expanded. After a week in culture, sorted endothelial cells displayed typical “cobblestone” morphology exhibited by cultured primary and immortalized endothelial cell lines. Fluorescence microscopy of the MACS purified hiPSC-ECs demonstrated lineage-specific expression of CD31, Enos, VECAD, vWF and live Ac-LDL uptake for Wnt, SMAD, and MEK/MAPK signaling pathways. The hiPSC-ECs also formed typical cord-like structures, stable for up to 48 h in a standard Matrigel angiogenesis assay. Flow cytometry analysis demonstrated the population at the end of differentiation on day 5 was 33% CD31 + , 58% VEGFR2 (KDR) + , and 68% VE-CAD. The overall differentiation efficiency for CD31 expression with this method was determined to be 27.8% ± 4.6% with flow cytometry analysis of four different differentiations on separate occasions. The purified population after two passages was 92% CD31 + , 78% VEGFR2 (KDR) + , and 99% VECadherin + . Temporal gene expression analysis demonstrated the expression of pluripotency markers OCT4 and NANOG at day − 1 and no expression by day 5. Accordingly, the upregulation of early mesoderm marker T (Brachyury) was observed 24 h after differentiation was initiated and little expression was detected by day 5. Increased gene expression for endothelial cell lineage markers CD34, PECAM1 (CD31), ENG (CD105), KDR (VEGFR2), FLT1 (VEGFR1), and CDH5 (VE-CAD) was observed by day 5. The characterization of the hiPSCs demonstrates endothelial cell-specific gene expression, phenotype, and stable network formation within HyA-based hydrogels, indicating the hiPSC-ECs' suitability for assessing the angiogenic potential of novel biomaterials.

Endothelial cells (EC) and pericytes have been differentiated from keratinocyte-derived hiPSCs [36]. This protocol includes an additional 3D embryoid body (EB) stage which substantially enhances the differentiation efficiency in comparison to the previously described monolayer-based differentiation protocol [37]. The protocol starts by culturing the cells for one day in an aggregation medium and ROCK inhibitor to create aggregates. After, cells were cultured in N2B27 media supplemented with CHIR99021 and BMP-4 to allow the induction of mesoderm. On Day 3, the media was changed to N2B27 medium containing VEGF-A and forskolin. After 5 days, aggregates were cut and cultured on gelatin-coated plates containing StemPro-34 medium supplemented with VEGF-A and FGF-2. On D8, the medium was replaced using the same medium as on day 5. CD31 + (ECs) and CD31- (pericytes) cells were then sorted and plated on gelatin-coated plates in EC and Pericyte medium supplemented with VEGF-A, FGF-2, and Y-27632. Co-culturing these cells for 7 days in EC and Pericyte media supplemented with VEGF-A, FGF-2, and the transforming growth factor beta (TGF-β) inhibitor SB431542 forms vessel-like structures under 2D cell culture conditions. In addition, these EC and pericytes cells cultured in a 3D fibrin gel supplemented with VEGF-A and FGF-2 for 7 days were able to form a 3D vascular network in the AIM Biotech 3D cell culture chip, therefore, cells that are differentiated using this novel approach can fulfill their main function [36]. For this reason, incorporating these cells into a microfluidic chip can successfully create a 3D microvascular network, meaning that these can feasibly be used in future microfluidic research.[36].

Significant progress has been made in directed differentiation protocols for human pluripotent stem cell monolayers to endothelial cell lineage. However, many issues remain with total differentiation time, the use of several different culture media, and late stage expression of mature endothelial cell markers. Total differentiation time needs to be decreased since most protocols consist of a 5–10 day differentiation process with an additional maturation phase that can last up to 60 days before the ECs can be used in a functional capacity. Additionally, current protocols entail the use of more than one media formulation, including MEF-conditioned media, from the pluripotency stage to the differentiation and maturation process. Considering the culture and maintenance of the pluripotent stem cells before differentiation, the total process can take up to 3 months with 2–3 different media formulations. Finally, many protocols report the expression of mature EC markers such as CD31 and VE-Cadherin on the final day of differentiation or after the maturation phase.

## Human stem cell-derived 3D in vitro angiogenesis models with engineered ECM biomaterials and microfluidic systems for preclinical drug screening applications

**Fig. 1:**
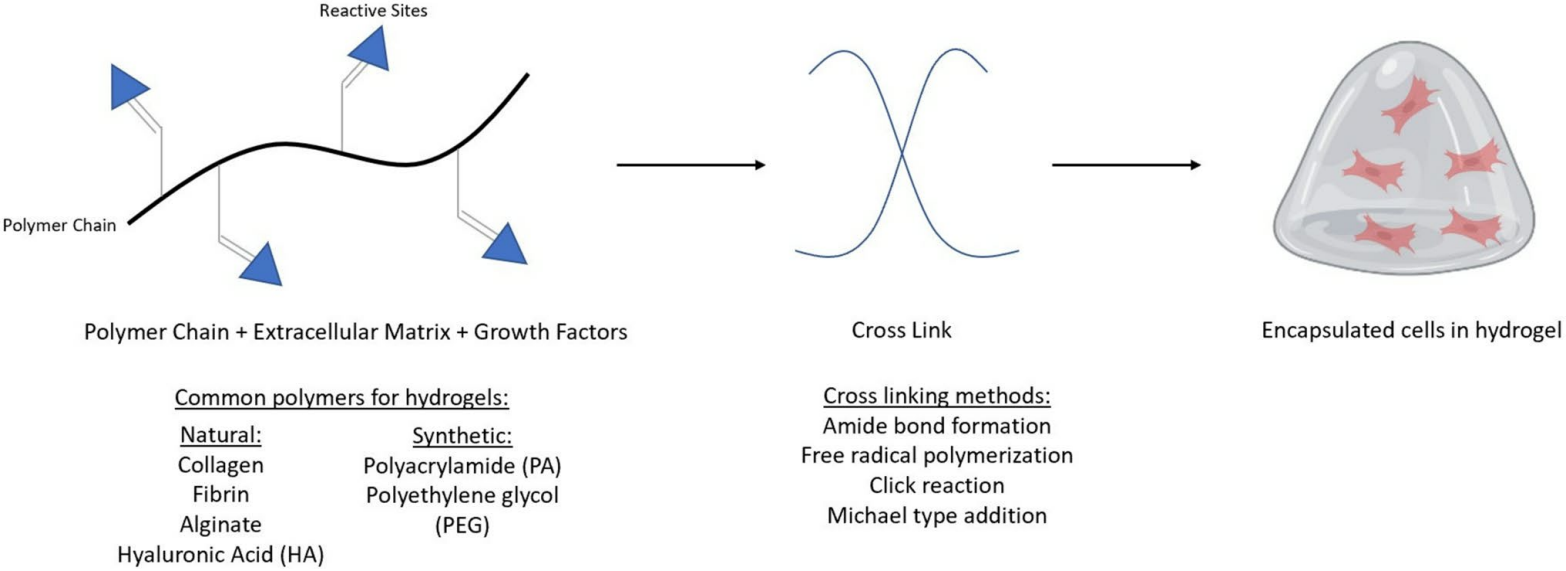
Schematic overview of common natural and synthetic hydrogel-forming biomaterials that can be used to encapsulate hiPSC-ECs to form 3D Angiogenesis tissue models [7–24]

**Fig. 2:**
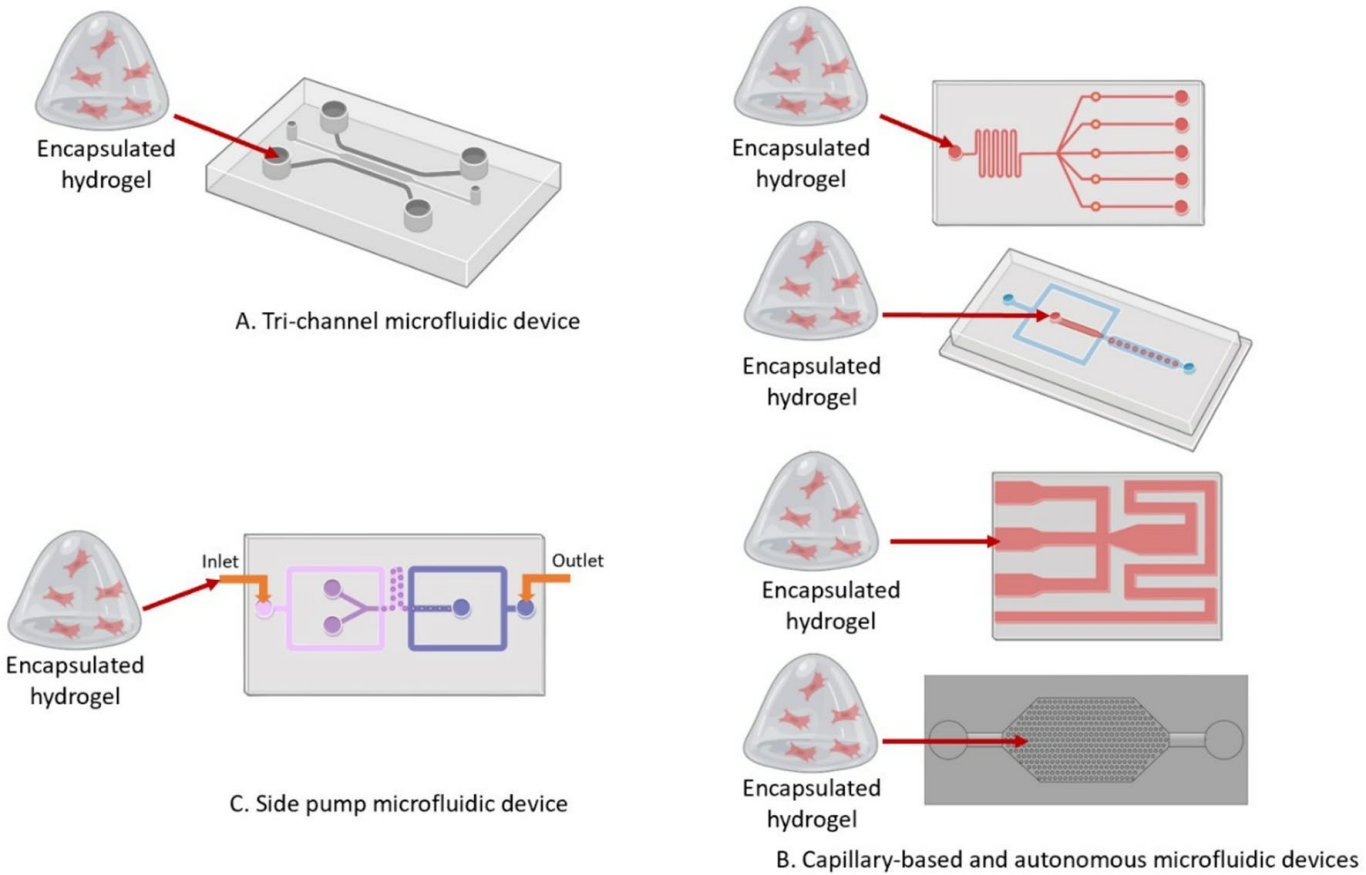
Common microfluidic device designs and associated sample loading that promote capillary-like tube formation of 3D Angiogenesis models **a** Tri-channel **b** side pump **c** capillary circuit [37–45]

**Fig. 3:**
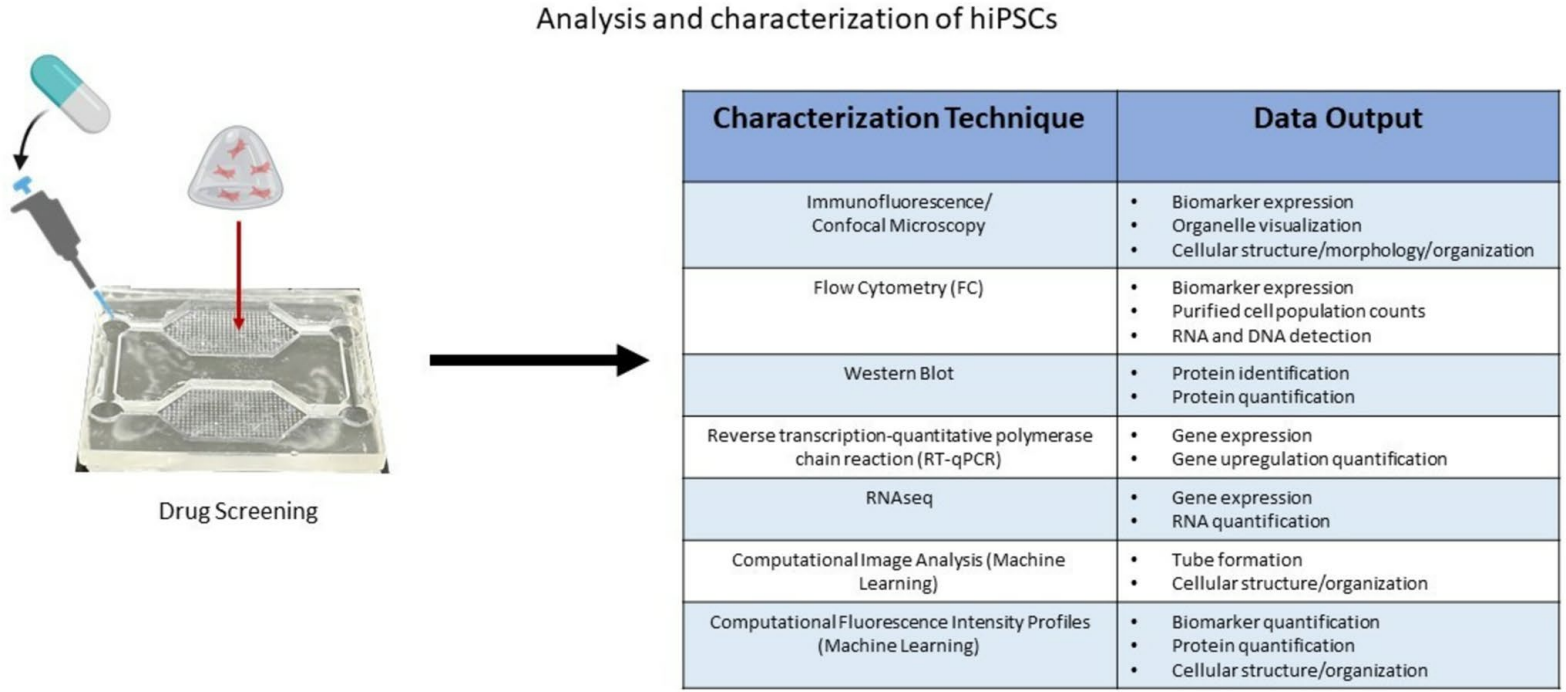
Drug screening studies can be applied to hPSC-derived 3D Angiogenesis models cultured within dynamic microfluidic platforms and analyzed with standard characterization techniques and data output [18, 25, 27, 30, 37–39]. (closed-circuit capillary-flow microfluidic device developed in Natividad-Diaz Lab shown)

The advancement of microfluidic device designs has facilitated the increased complexity of physiologically-relevant 3D in vitro angiogenesis models [23, 25, 27, 38–47]. The general process for making 3D in vitro angiogenesis models involves the encapsulation of hPSC-derived endothelial cells in a biomaterial (natural or synthetic) (Fig. 1) and dynamic culture within a microfluidic platform (Fig. 2) [25, 27, 40–44, 46, 48–52]. The biomaterial should provide a supportive environment similar to the native ECM that will stimulate cell viability, proliferation, and function [21, 34, 53–59]. Natural protein ECM materials have inherent cell adhesion and growth factor binding sites which support encapsulated cell function and viability [60]. Examples of natural protein ECM biomaterials that support angiogenesis include collagen, gelatin, fibrin, vitronectin, fibronectin, and Matrigel [54, 58, 61–71]. These natural biomaterials allow biomimetic cell–matrix interactions and ECM remodeling similar to in vivo mechanisms. [72, 73]. Polysaccharide-based hydrogels such as hyaluronic acid and alginate can be chemically modified to support endothelialcell adhesion and growth factor sequestration to promote capillary-like network formation in vitro [54, 58, 65, 69, 74–80]. However, natural ECM biomaterials have low mechanical strength, batch to batch variability, quicker degradation times, and their microarchitecture may be affected by concentration, temperature, and pH, which may affect reproducibility and control of experimental parameters [73, 81]. Synthetic biomaterials consist of artificially derived substrates which are typically less bioactive compared to natural ECM materials [72, 81]. Synthetic ECM biomaterials can be functionalized with cell adhesion peptides, chemically modified to have tunable mechanical properties such as tissue specific-stiffness, and crosslinked with enzymatically degradable biomolecules for cell remodeling of the ECM [72, 73]. Another advantage of synthetic ECM biomaterials is they can be precisely manufactured and reproduced for minimal variability for control over material stiffness and degradation. Synthetic ECM biomaterials that promote angiogenesis and capillary-like network formation in vitro include polyacrylamide (PA) and polyethylene glycol (PEG). These synthetic biomaterials offer more tunable chemical and mechanical properties relative to natural biopolymers, but have issues with matrix degradation and byproduct toxicity [27, 82–86]. Figure 1 summarizes some of the common biomaterials used to encapsulate hiPSC-EC for development of 3D in vitro angiogenesis models. Many review papers provide more in-depth discussion about the relationship between chemical and mechanical properties for these ECM biomaterials and their influence on cell function and structural organization [16, 62, 68, 76, 80, 87–89]. Microfluidic devices provide a controlled, reproducible environment for the dynamic culture of 3D in vitro Angiogenesis models [23, 25, 27, 35, 38, 43, 46, 90, 91]. Microfluidic device designs consist of multiple microchannels interconnected with tissue culture chambers [7, 25, 38, 47, 49, 92–94]. Fluid flow within these platforms can be controlled with externally applied pressure, such as a syringe pumps or driven autonomously through capillary action [95–97]. Microfluidic device design can be completed using widely available computer aided model (CAD) software (Solidworks, Fusion, AutoCAD, Shapr3D) and fabrication may include photolithography and/or 3D stereolithography printing for high-throughput manufacturing [82, 98]. Photolithography consists of generating silicon and photoresist molds over which a liquid set polymer such as PDMS is poured and cured [27]. The cured polymer is peeled from the mold surface and bonded to a glass slide to form a closed channel. General techniques for photolithography have created high resolution replica devices ranging from a few microns to hundreds of nanometers and intricate 3D geometries [99]. More rapid production can be done with 3D stereolithography (SLA) printing which uses a light-emitting diode (LED) to build a printed part layer by layer and is useful for creating fine features rapidly [99]. After fabrication, the device can be sterilized, and the 3D-engineered tissue construct can be loaded or placed into the device culture chambers. The enclosed system can then be placed into a cell culture incubator and observed over a desired experimental time period with regular media changes [35, 38, 43, 46, 90–92, 98, 100, 101]. Figure 2 summarizes the common microfluidic device designs used to encapsulate hiPSC-EC for development of 3D in vitro angiogenesis models. Following cell encapsulation into a biomaterial, several characterization techniques and analyses can be conducted to extract data from the angiogenesis model (Fig. 3). In this section, several ECM biomaterials and microfluidic systems that have been used to develop 3D in vitro angiogenesis models will be discussed. Although the overall scope of this review is to discuss the Implementation of hPSC-derived endothelial cells to develop more physiologically relevant 3D in vitro angiogenesis models, it is noteworthy to mention the biomaterials and microfluidic platforms in these studies are amenable to incorporation of commercially available or primary endothelial cells which have the capacity to remain viable and form capillary-like networks within these systems.

One of the earliest and most complex 3D in vitro vascular tissue models consisted of two fluidic channels separated by a central channel of 12 consecutively connected diamond tissue chambers [46]. The fluidic channels are connected on either side of the tissue chamber via a single connecting pore and fabricated using photolithography and polydimethylsiloxane (PDMS) techniques. Two design variations for connecting the fluidic channels and microtissue chambers were investigated to determine their effect on the vascular tissue formation using normal lung human fibroblasts (NHLFs) and human endothelial colony forming cell-derived ECs (ECFC-ECs) encapsulated in a fibrin hydrogel. One design (VP) consisted of connecting the arterial microfluidic line (high pressure side) from the last microtissue chamber directly to the venular side (low pressure side) of the same chamber. This design created a similar mean pressure within the microtissue chambers and a negligible velocity in the x-direction. However, a wide variation in the pressure drop in the y-direction and interstitial velocity across the microtissue chambers was created. The second design (EQ), consisted of connecting the arterial microfluidic line from the last microtissue chamber to the venular side of the first chamber. This design created a larger pressure drop across the microtissue chambers in the y-direction and a large but constant pressure drop in the x-direction.

Additionally, Biendarra-Tiegs et. al developed an in vitro model that recapitulates key features of in vivo atrial conduction. First, in vivo and ex vivo measurements of conduction velocity (CV) and effective refractory period (ERP) in adult human hearts were used to establish appropriate benchmarks for physiological relevant levels. A linear spiral device was microfabricated using PDMS to create a model system of human atrial conduction using human induced pluripotent stem cell-derived atrial-like cardiomyocytes (hiPSC-aCMs). hiPSC-aCMs were allowed to attach overnight and underwent depolarization and repolarization events. This model was viewed under a standard epifluorescent microscope and produced a CV that mimicked the in vivo and ex vivo measurements of human hearts. This study modeled atrial conduction with hiPSC-aCMs more accurately compared with 2D monolayers [102].

A microfluidic device to study tumor behavior and macrophage mechanisms in the tumor microenvironment was created with PDMS and consisted of three tissue chambers parallel to each other with microporous walls. Using patient-derived tumor cell lines from pancreatic ductal adenocarcinoma (PDAC) and colorectal cancer (CRC), macrophages with predominant M1 phenotype created an antitumor microenvironment which inhibited tumor growth, migration and angiogenesis. Macrophages with predominant M2 phenotype created a protumor microenvironment that stimulated tumor growth, migration and angiogenesis. Two distinct endothelial cell phenotypes were created in the presence of M1 and M2 macrophages and tumor cells. The tumor microenvironment reflected stable vessels and active angiogenesis, recapitulating importing features of tumor and vascular microenvironments [103]. A platform designed for multiple vascularized micro-organs (VMOs) to fit on a 96-well plate allowed each VMO to be independent from the next and function without the need for external pumps or valves [104]. The microfluidic platform was designed to fit a standard 96-well plate consisting of two layers, a middle layer with 12 microfluidic device units and a bottom layer with a thin transparent polymer membrane. The microfluidic device units are composed of 3 tissue chambers connected to 2 adjacent microfluidic channels, 2 gel loading ports, 2 medium ports and one pressure regulator unit. The platform, including both layers, was fabricated using photolithography and PDMS. Phan and Wang created vascularized microtumors (VMTs) and in a blinded screen, assayed the VMTs with FDA-approved anti-cancer drugs. Human endothelial colony-forming cell-derived endothelial cells (ECFC-EC) were expanded on fibronectin-coated flasks and cultured in EGM-2. Human normal lung fibroblasts (NHLF) were cultured in DMEM. VMTs were allowed to develop for 7 days before drug exposure. Each unit with VMO or VMT was exposed to standing primary screening of 1 uM. The compounds were dissolved in dimethyl sulfoxide (DMSO) and diluted into the cell culture medium. Tissues were exposed to the compounds for 72 h before quantifying the effect on total vessel length and tumor growth. The FDA-approved anti-cancer compounds were: bortezomib, vincristine, CP-673451, linifanib, tamoxifen, axitinib, sorafenib, mitomycin C, vorinostat, and gemcitabine. The negative control compounds were isoprenaline and propranolol. The control compounds showed no effect on either tumor growth or vasculature. Bortezomib and linifanib effectively targeted tumor growth and vasculature. Tamoxifen, mitomycin C, gemcitabine, and vorinostat were more effective in targeting tumor growth, while vincristine and axitinib showed preferential effects on targeting the vasculature. Long-term treatment times that targeted the vasculature also reduced the tumor growth due to the reduced nutrient supply. Here, the standardized platform to fit into a 96-well plate makes larger-scale drug screening simple and negated the need for external pumps.

To establish a physiologically relevant in vitro angiogenesis inhibition screening assay, researchers used human umbilical vein endothelial cells (HUVECs) and iPSC-ECs seeded into microfluidic channels coated with fibronectin adject to a patterned collagen-I hydrogel.[105]. A platform with 40 individual microfluidic units patterned underneath a 384-well plate. A single microfluidic unit or chip consists of 3 channels, a central gel channel and two perfusion channels. ECs were cultured for 2 days in a medium supplemented with vascular endothelial growth factor-165 and bFGF to form confluent microvessels. An angiogenic sprouting mixture was prepared by supplementing HE-SFM with phorbol 12-myristate 12-acetate and sphingosine-1-phosphate. The mixture was added to the inlets of the device. The model was evaluated by placing patterned collagen-1 hydrogels inside of the devices and seeding the cells in the fibronectin coated channel. After two days, confluent iPSC-ECs microvessels formed and were subjected to angiogenic growth factors for 2 additional days. Concentration for sunitinib inhibition was optimized, where sprouting occurred at concentrations > 10 nM, while angiogenesis was completely inhibited at concentrations > 50 nM. The inhibition sprouting of iPSC-ECs with primary HUVECs was compared using sunitinib and 3PO. Sprouting length reduction at 50% is achieved at concentrations around 20 nM for iPSC-ECs and 66 nM for HUVECs. However, HUVECs showed single-cell migration into the collagen-I at high concentrations, whereas the sprouting of iPSC-ECs was completely blocked. These results showed that 3PO inhibits the angiogenic sprouting of iPSC-ECs and like HUVECs, they undergo the same metabolic switch to use glycolysis as the main energy source during angiogenic sprouting. It was demonstrated that iPSC-ECs can be used in a physiologically relevant in vitro angiogenesis inhibition screening assay.

Two in vitro models of brain angiogenesis were developed using human umbilical vein endothelial cells (HUVECs) and iPSC-derived brain microvascular endothelial cells (dhBMECs) [106]. The beads were coated with collagen and incubated in three media conditions: basal media, basal media supplemented bFGF, and basal media supplemented with bFGF, VEGF and Wnt7a. When embedded into collagen 1 hydrogels, there was low angiogenic behavior in the absence of growth factors, whereas the addition of VEGF and Wnt7a produced a higher angiogenic phenotype. BBB beads cultured in VEGF and wnt7a showed extensive networks of angiogenic sprouts and formation of lumen-like structures. Four ECM compositions were studied with collagen, Matrigel, and fibrin and it was revealed changing the ECM composition had a small effect on angiogenic phenotype across the conditions, although Matrigel supplementation led to increase sprouting, but was not significantly different. To model pathological brain angiogenesis, the BBB beads in collagen 1 were supplemented with Matrigel and subjected to high and low concentrations of hydrogen peroxide (H2O2) in the absence of external growth factor stimuli. After two days of exposure to H2O2, the formation of sprouts demonstrated an increased angiogenic phenotype and over 3 days allowed for assembly into BBB microvessels.

## Discussion and conclusions

Angiogenesis and vascular dysfunction are fundamental components for life-threatening illnesses, including cardiovascular disease, cancer tumor growth and metastasis. In vitro angiogenesis models provide controlled, and reproducible platforms for therapeutic drug screening along with fundamental molecular and cellular studies for specific patient populations when combined with hPSC technology. Current directed differentiation protocols for human pluripotent stem cells to endothelial cells, have demonstrated the expression of mature lineage-specific markers with differential arterial and venous sub-types. Recent studies on 3D in vitro Angiogenesis models with microphysiological systems and microfluidic devices have demonstrated self-assembly into vascular networks with physiologically-relevant metabolic responses and pharmacokinetics to exogenously added biochemicals/drugs. Within these models, drug screening, patient-specific source variation, and regeneration of tissues can be studied in more detail within a controlled microenvironment. Table 2 summarizes the advantages/disadvantages of the most commonly used endothelial cell types, extracellular matrix biomaterials, and microfluidic device fabrication techniques to develop 3D in vitro human angiogenesis models.

**Table 2:** Advantages/Disadvantages associated with commonly used Endothelial cell types, Extracellular Matrix Biomaterials, and Microfluidic device fabrication techniques to develop Human 3D in vitro Angiogenesis Models

	Common subtypes or techniques	Advantages	Disadvantages	User skill level
Cell type [18, 21, 22, 33–36, 106]	Human induced pluripotent stem cell derived endothelial cells (hiPSC-ECs)	• More physiologically relevant • Potential for patient-specific model • Lineage-specific biomarker expression and function	• Differentiation protocols are diverse with varying efficiency • Potential presence of oncogenic gene expression • Advanced user training and skill level required	+ +
	Human umbilical vein endothelial cells (HUVECs)	• Well established cell line used to study normal EC behavior and response to stimuli and/or treatment • Does not require differentiation	• Limited number of cell passage in culture • Limited potential for patient-specific model	+
	Human endothelial colony-forming cell-derived endothelial cells (ECFC-EC)	• Well established cell line used to study normal EC behavior and response to stimuli and/or treatment • Used to study endothelial dysfunction and vascularization	• Limited number of cell passage in culture • Limited potential for patient-specific model	+
	Human tissue-specific microvascular endothelial cells (HMVEC-brain, HMVEC-Cardiac, HMVEC-Lung, HMVEC-Dermal)	• More physiologically relevant form of modeling human tissue-specific endothelial cells • Characterized by expression of representative tissue-specific biomarkers and function	• Limited number of cell passage in culture • Limited potential for patient-specific model	+
Extracellular Matrix (ECM) Biomaterial [27, 54, 58, 61–71, 74–86]	*`Natural*			
	Collagen	• Good inherent adhesion and cell migration support • Biocompatible • Supports vascularization and angiogenesis • Higher mechanical strength for natural ECM materials	• Batch to batch variability • Quick degradation	+
	Fibrin	• Good inherent adhesion and cell migration support • Biocompatible • Supports vascularization and angiogenesis	• Batch to batch variability • Low mechanical strength • Quick degradation	+
	Matrigel	• Good inherent adhesion and cell migration support • Biocompatible • Supports vascularization and angiogenesis • Supports attachment and growth of human stem cells	• Batch to batch variability • Limited composition control • Derived from mouse tumor cells • Low mechanical strength • Quick degradation	+
	Hyaluronic acid (HA)	• Promote cell proliferation and wound healing • Amenable to chemical modification	• Requires chemical modification for cross-linking and cell adhesion peptides • Low mechanical strength • Quick degradation	+
	*Synthetic*			
	Poly(ethylene glycol) (PEG)	• Tunable mechanical properties • Biocompatible	• Need to be modified to allow specific cell adhesion peptides • Limited degradation and clearance	+
	N-isopropylacrylamide (NIPAAM)	• Thermosensitive • Biocompatible	• Need to be modified to allow specific cell adhesion peptides • Limited degradation and clearance	+
Microfluidic device fabrication [27, 37, 99]	Photolithography	• Nanometer-scale resolution • Complex device design	• Can take up to several days to complete • Requires clean room	+ +
	3D Printing	• Rapid production • Complex device design	• Printing resolution may vary • Micrometer-scale resolution for most commercially available 3D printers • Batch to batch variability	+

+ standard laboratory training,  +  + advanced laboratory training required

The recent growth and establishment of hPSC and organoid biorepositories worldwide have improved the potential large-scale use of patient-specific 3D in vitro angiogenesis models for fundamental and drug screening/discovery research. Current biorepositories are storage facilities for biological materials, including animal and human tissue samples, which are critical to improving and progressing the development of targeted diagnostics and therapies for patients. Advanced Technologies for the Preservation of Biological Systems (ATP-BIO) works to increase the storage capacity and transportation of cells, aquatic embryos, tissue, skin, whole organs, microphysiological systems, and whole organisms by utilizing advanced biopreservation technologies [107]. At the Coriell Institute for Medical Research, the NIGMS repository contains over 11,000 cell lines, 5900 DNA samples, and over 85 iPSC lines, and including their repository samples that represent different disease states, chromosomal abnormalities, and distinct human populations [108]. The National Disease Research Interchange (NDRI) provides human organs and tissue from diverse normal and diseased donors to support the different experimental needs of biomedical research projects [109]. The European Bank for induced pluripotent Stem Cells (EBiSC) banks and distributes iPSC lines to develop improved stem cell culture, cryopreservation systems, and provide accessible products used to advance medicine development [110]. These biorepositories serve as resources to obtain patient-specific biological samples to develop physiologically relevant 3D in vitro angiogenesis tissues and models. Angiogenesis models developed with patient-specific cells into 3D tissues can have the potential to be biopreserved and stored in biobanks. Furthermore, biobanks can store the biological samples within these models and become a resource for researchers worldwide. Researchers can request specific samples and tissues to investigate physiologically relevant processes and drug screening.

## Abbreviations

CVD: Cardiovascular disease
hPSC: Human pluripotent stem cells
hiPSCs: Human induced pluripotent stem cells
ECs: Endothelial cells
EVCs: Early vascular lineage cells
FBS: Fetal bovine serum
ECGM: Endothelial cell growth medium
VEGF: Vascular endothelial growth factor
hPSCs: Human pluripotent stem cells
BMP4: Bone morphogenetic protein 4
ITS-X: Insulin-transferrin-selenium-ethanolamine
MTG: Mono thiol glycerol
TGF: Transforming growth factor
HUVECs: Human umbilical vein endothelial cells
EPCs: Endothelial precursor cells
AELCs: Arterial endothelial-like cells
HUAECs: Human umbilical artery endothelial cells
VELCs: Venous endothelial-like cells
PLLA: Poly (L-lactic acid)
PCL: Poly (caprolactone)
PDMS: Poly (dimethyl siloxane)
SFM: Serum free medium
bFGF: Basic fibroblast growth factor
EGF: Epithelial growth factor
AECs: Arterial endothelial cells
VECs: Venous endothelial cells
ECM: Extracellular matrix
PA: Poly acrylamide
PEG: Polyethylene glycol
CAD: Computer aided design
SLA: Stereolithography
LED: Light emitting diode
CV: Conduction velocity
ERP: Effective refractory period
hiPSC-aCMs: Human induced pluripotent stem cell-arterial like cardiomyocytes
PDAC: Pancreatic ductal adenocarcinoma
CRC: Colorectal cancer
VMOs: Vascularized micro-organs
VMTs: Vascularized micro-tumors
ECFC-ECs: Endothelial colony forming cell derived endothelial cells
NHLF: Human normal lung fibroblasts
DMSO: Dimethyl sulfoxide
dhBMECs: Human induced pluripotent stem cell derived brain microvascular endothelial cells
BBB: Blood brain barrier
Hydrogen peroxide: H_2_O_2_
